# Preliminary Results of a 3D‐Printed Modular Vertebral Prosthesis for Anterior Column Reconstruction after Multilevel Thoracolumbar Total En Bloc Spondylectomy

**DOI:** 10.1111/os.12975

**Published:** 2021-04-04

**Authors:** Xiaodong Tang, Yi Yang, Jie Zang, Zhiye Du, Taiqiang Yan, Rongli Yang, Wei Guo

**Affiliations:** ^1^ Musculoskeletal Tumor Center Peking University People's Hospital Beijing China

**Keywords:** Spinal tumor, En bloc, Spondylectomy, 3D‐printed, Vertebral prosthesis

## Abstract

**Objective:**

To evaluate preliminary results of a 3D‐printed modular prosthesis for spinal reconstruction after multilevel thoracolumbar total en bloc spondylectomy (TES).

**Methods:**

Patients with thoracolumbar spinal tumors treated surgically between January 2016 and April 2019 were included in this retrospective study. A total of 17 male and 10 female patients with a mean age of 42 (range, 15–72) years comprised the sample. The pathological diagnoses included six chondrosarcomas (one of them was mesenchymal chondrosarcoma), six giant cell tumors, three malignant peripheral nerve sheath tumors, two osteosarcomas, two undifferentiated high‐grade pleomorphic sarcomas (UPS), two solitary fibrous tumors, one Ewing's sarcoma, one liposarcoma, and four metastatic tumors. Tumors involved 2 levels in 14 patients, 3 levels in seven patients, 4 levels in four patients, 5 levels in one patient, and 6 levels in one patient. A 3D‐printed modular prosthesis was used for anterior column reconstruction after TES. All analyses were performed using SPSS version 18.0 (SPSS, Inc., Chicago, IL). Descriptive statistics were used to analyze the demographic data and clinical outcomes. Data forms included mean, standard deviation and range.

**Results:**

Under general anesthesia, all patients received TES with an average operative time of 639 (range, 210–1650) min, and the mean blood loss during operation was 4.1 (range, 0.8–13.3) L. Twenty‐two patients have been transferred to ICU for a mean time of 3.2 (range, 0–6) d. All patients had follow‐up procedures except for one, who died of perioperative complications. Mean time of follow‐up was 22 (range, 12–41) months. Local recurrence (19.2%) occurred in two patients with intralesional margin and three patients with marginal margin, respectively. At the end of follow‐up, three patients died of disease, eight patients were alive with disease, and 15 patients had no evidence of disease. Average lengths of resected vertebrae and modular prostheses were 71.4 ± 26.5 mm (range, 40–142 mm) and 68.4 ± 23.9 mm (range, 40–132 mm), respectively. In 26 patients with minimum follow‐up of more than 1 year, no evidence of internal fixation failure or dislocation of vertebral prosthesis was found. Asymptomatic prosthetic subsidence into adjacent vertebral bodies occurred in 10 patients with a mean length of 1.8 ± 1.0 mm (range, 1–4 mm). The subsidence was seen at proximal end in two patients, distal end in four patients, and both ends in four patients. Eighteen major complications and 14 minor complications were found in 15 patients. All patients fully recovered at 3 or 6 months after operation. At the latest follow‐up, in 23 alive patients, 19 can walk independently and two can achieve outdoor activities by walking aid.

**Conclusion:**

For spinal reconstruction after multilevel thoracolumbar TES, 3D‐printed modular vertebral prosthesis is suitable for different length of anterior column reconstruction with less mechanical complications, and can provide a stable environment to maintain or rehabilitate patients' neurological function in short‐term follow‐up.

## Graphical abstract

3D‐printed modular vertebral prosthesis for spinal reconstruction after multilevel thoracolumbar TES is suitable for different lengths of anterior column reconstruction with less mechanical complications, and can provide a stable environment to maintain or rehabilitate patients' neurological function in short‐term follow‐up.

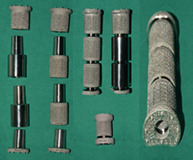

## Introduction

Total en bloc spondylectomy (TES) has been proven as an effective treatment for aggressive benign, primary malignant, and solitary metastatic spinal tumors^1^, ^2^, ^3^. Owing to the complete disruption of the spinal connection, reconstructions including vertebral body replacement and posterior pedicle screws and rods fixation after TES are challenging and have a high incidence of instrument failure^4^. Multilevel TES is more complicated and has a higher complication rate compared with single‐level resection^5^, ^6^. Spinal instability is influenced by the number of segments that need to be removed.

Widely used anterior column reconstruction techniques include vascularized or nonvascularized autograft strut, structural allograft strut, cage systems (titanium, expendable titanium, stackable carbon cages), and PMMA^7^, ^8^, ^9^, ^10^. For single‐level vertebral defects, titanium mesh cages or expandable cages are sufficient for anterior column reconstruction^9^. While in reconstruction of multilevel spinal defects, deficiencies of various methods including limited amount of autograft structural bone, nonunion and fracture of allograft, high incidence of subsidence of mesh cages, insufficient length of using expandable cage, and high cost of carbon stackable cages should all be considered.

Three‐dimensional (3D)‐printed implants have recently been introduced into orthopaedic surgery, and have shown excellent capacity of bone ingrowth and biological fixation at metal–bone interface^11^, ^12^. A few reports have been done on custom‐made 3D‐printed vertebral prosthetic replacement after spondylectomy for patients with spinal tumor^13^, ^14^, ^15^. Optimal results have been seen from case reports and a small number of patients with short‐term follow‐up. However, these custom‐made prostheses have drawbacks, including a period of waiting time for production and difficulty of reconstruction in case of a mismatch between the resected specimen and the prosthesis^13^. A stable anterior column reconstruction after multilevel TES should not only provide osseous fusion and avoid hardware failure, but also adapt to a different range of spinal bone defects. To solve these problems, we designed a system of 3D‐printed modular vertebral prosthesis for replacement after TES, especially in multilevel patients.

**Fig. 1:**
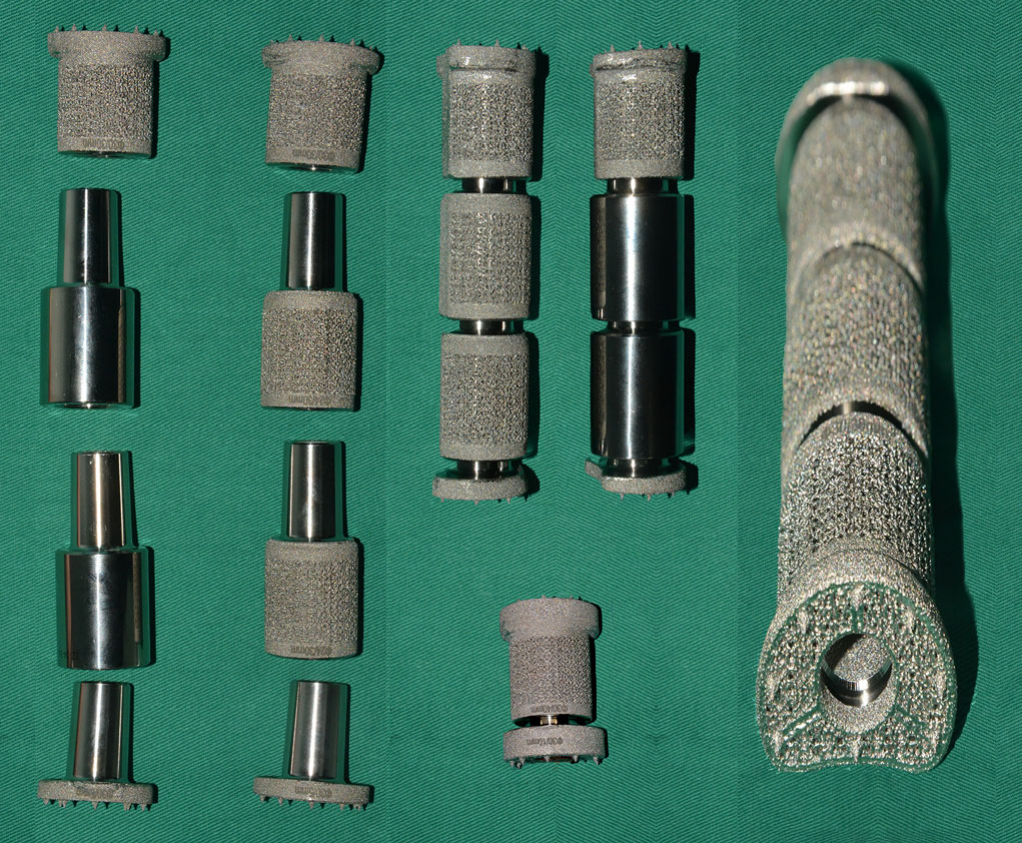
The 3D‐printed modular vertebral prostheses are shown in combination with superior endplate, inferior endplate, and different body segments. The endplate component is designed with an electron beam melting manufactured porous surface and a bone graft hole.

The 3D‐printed modular vertebral prosthesis (AK MEDICAL HOLDINGS LIMITED, Beijing, China) which is designed by the senior author (WG) was prepared for reconstruction of anterior column preoperatively (Fig. 1). This prosthesis made by Ti6Al4V is composed of a superior endplate, an inferior endplate, and body segments with different sizes. The modular junctions of components are based on a male–female taper fit. Through the appropriate matching process, vertebral prosthesis can be assembled with different lengths (25–200 mm with a minimum interval of 2.5 mm), diameter (21, 24, 30 mm), and degree of curvature (0°, 4°, and 8°). The endplate component has a porous surface produced by electron beam melting. This surface permits bone ingrowth and arthrodesis, and several tiny sharp conical projections on the porous surface can provide additional early fixation with adjacent vertebrae. Two kinds of body segment with porous surface or polished surface can be selected. An empty hole which permits bone graft is designed in the middle of prosthesis to facilitate osseous union.

The purpose of this study is to evaluate the preliminary results of this 3D‐printed modular vertebral prosthesis for spinal reconstruction after multilevel thoracolumbar TES. This study aims to determine if this prosthesis: (i) is suitable for different lengths of reconstruction; (ii) has less mechanical complications; (iii) and can provide a stable environment to maintain or rehabilitate patients' neurological function in a short‐term follow‐up.

## Patients and Methods

### Patient Demographics

This study was approved by the hospital IRB committee. Patients with spinal tumors treated surgically between January 2016 and April 2019 were reviewed retrospectively. The inclusion criteria included: (i) patients with aggressive benign or malignant tumors in their thoracolumbar spine; (ii) patients who received TES at two or more levels; and (iii) patients who had the replacement with a 3D‐printed modular vertebral prosthesis. The exclusion criteria included: (i) patients with piecemeal excisions; (ii) patients with partial vertebrectomy; and (iii) patients with other methods for anterior column reconstruction.

Finally, 27 patients with a mean age of 42 (range, 15–72) years were included in this study (Table 1). A total of 17 male and 10 female patients comprised the sample. The pathological diagnoses included six chondrosarcomas (one of them was mesenchymal chondrosarcoma), six giant cell tumors, three malignant peripheral nerve sheath tumors, two osteosarcomas, two undifferentiated high‐grade pleomorphic sarcomas (UPS), two solitary fibrous tumors, one Ewing's sarcoma, one liposarcoma, and four metastatic tumors. The tumors involved 2 levels in 14 patients, 3 levels in seven patients, 4 levels in four patients, 5 levels in one patient, and 6 levels in one patient. Adjuvant therapies including chemotherapy, radiation, and targeted therapy were carried out in patients with osteosarcoma, chondrosarcoma, malignant peripheral nerve sheath tumor, UPS, and metastatic tumor. Preoperatively, all patients were evaluated using the Weinstein–Boriani–Biagini surgical staging system^16^.

**TABLE 1:** Patients’ demographic data

No.	Age (yr)	Sex	Diagnosis	Involved levels	WBB	Adjuvant treatments	Follow‐up time (month)	Local recurrence	Survive	Frankel grade	
										Preoperative	Postoperative
1	52	M	Chondrosarcoma	T_3,4_	8‐12/B‐D	None	41	No	Ned	E	E
2	72	M	Chondrosarcoma	T_5‐7_	10‐5/A‐D	None	‐	‐	Dod	E	E
3	29	M	GCT	T_4,5_	11‐3/A‐D	Denosumab	36	No	Ned	E	E
4	53	F	Metastatic tumor	T_10‐12_	10‐4/A‐D	Radiotherapy	27	No	Dod	E	E
5	30	M	Chondrosarcoma	T_3‐6_	6–11/A‐D	Chemotherapy	34	Yes	Awd	C	C
6	43	M	Solitary fibrous tumor	T_11,12_	11‐7/A‐D	None	33	No	Ned	E	E
7	49	M	Metastatic tumor	T_4‐6_	8‐3/A‐D	Radiotherapy/Targeted therapy	30	Yes	Awd	A	A
8	47	M	Giant cell tumor	T_2‐4_	8‐2/A‐D	None	29	No	Ned	E	E
9	15	M	Osteosarcoma	T_11,12_L_1_	1‐8/B‐D	Chemotherapy	27	No	Ned	E	E
10	56	M	Metastatic tumor	T_3,4_	12‐5/A‐D	Radiotherapy	24	No	Dod	E	E
11	15	M	Osteosarcoma	T_12_L_1_	12‐7/B‐D	Chemotherapy	22	No	Awd	E	D
12	28	F	Chondrosarcoma	T_2,3_	11‐6/A‐D	None	21	No	Ned	E	C
13	52	M	Solitary fibrous tumor	T_8,9_	8‐2/A‐D	None	20	No	Ned	E	E
14	47	F	UPS	L_3,4_	11‐6/A‐C	Radiotherapy/Chemotherapy	19	No	Awd	E	E
15	66	F	Liposarcoma	T_8,9_	8‐4/B‐C	None	19	No	Ned	E	E
16	36	F	GCT	T_9‐12_	9‐2/A‐E	Denosumab	19	No	Ned	A	A
17	69	M	Metastatic tumor	T_7,8_	8‐2/A‐D	Radiotherapy/Targeted therapy	19	Yes	Awd	E	E
18	22	F	GCT	L_4,5_	10‐3/A‐D	Denosumab	19	No	Ned	E	D
19	37	M	Chondrosarcoma	T_1‐5_	7‐2/A‐D	Targeted therapy	17	Yes	Awd	E	D
20	34	F	Giant cell tumor	T_7‐12_	7‐3/A‐C	Denosumab	17	No	Ned	E	C
21	50	M	MPNS	L_1‐3_	9‐5/A‐D	None	17	No	Ned	E	E
22	58	M	Chondrosarcoma	L_2,3_	10‐3/A‐D	None	16	Yes	Awd	C	D
23	36	M	UPS	T_6‐9_	5‐11/A‐D	Chemotherapy/Radiotherapy	16	No	Awd	D	E
24	61	M	MPNS	T_1,2_	11‐6/A‐D	Targeted therapy	14	No	Dod	E	E
25	29	M	Ewing's sarcoma	T_6‐8_	11‐5/A‐C	Chemotherapy/Radiotherapy	16	No	Ned	E	E
26	30	F	MPNS	T_5‐8_	6–12/A‐D	Chemotherapy/Radiotherapy	14	No	Ned	E	E
27	27	F	GCT	T_8,9_	6‐1/A‐D	Denosumab	12	No	Ned	E	E

Awd, Alive with disease; Dod, Died of disease; GCT, Giant cell tumor; MPNS, Malignant peripheral nerve sheath tumor; Ned, No evidence of disease.

### Surgical Procedures

**Fig. 2:**
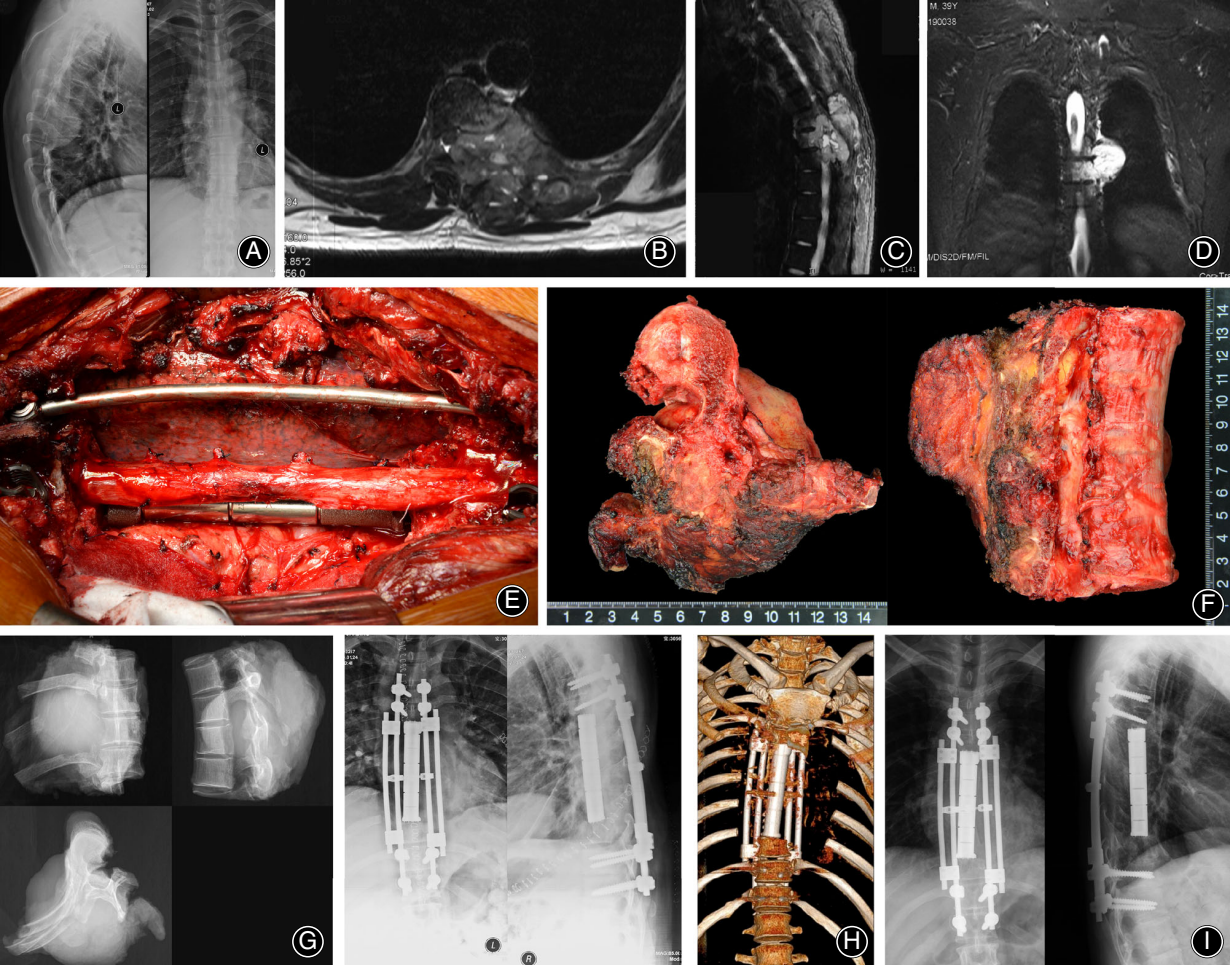
A male patient (No. 23) with spinal UPS underwent TES and reconstruction with the 3D‐printed modular vertebral prosthesis. (A) His preoperative plain radiograph and (B) MRI T2‐weighted axial view, (C) sagittal view, and (D) coronal view of images show involvement of T6‐T9 and left chest wall. (E) An intraoperative view shows the anterior column between T_5_ and T_10_ was reconstructed with a 3D‐printed modular vertebral prosthesis after removal of the tumor. (F) The gross view and (G) plain radiograph of the specimen show en bloc resection of T_6_‐T_9_ vertebrae. (H) Postoperative plain radiograph and reconstructive computed tomography show spinal reconstruction. (I) Plain radiograph at 12 months postoperative follow‐up shows intact internal fixation.

**Fig. 3:**
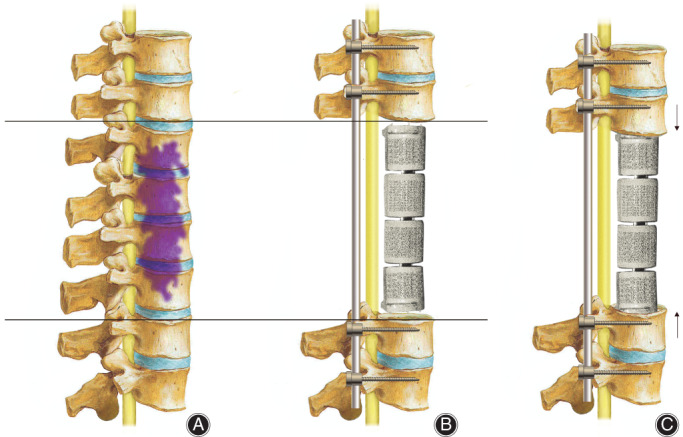
Diagrams for key points of the surgery: spinal reconstruction for a large defect. (A) En bloc spondylectomy. (B) Insertion of the 3D‐printed modular vertebral prosthesis. (C) Spinal shorten to grip the vertebral prosthesis tightly.

**Fig. 4:**
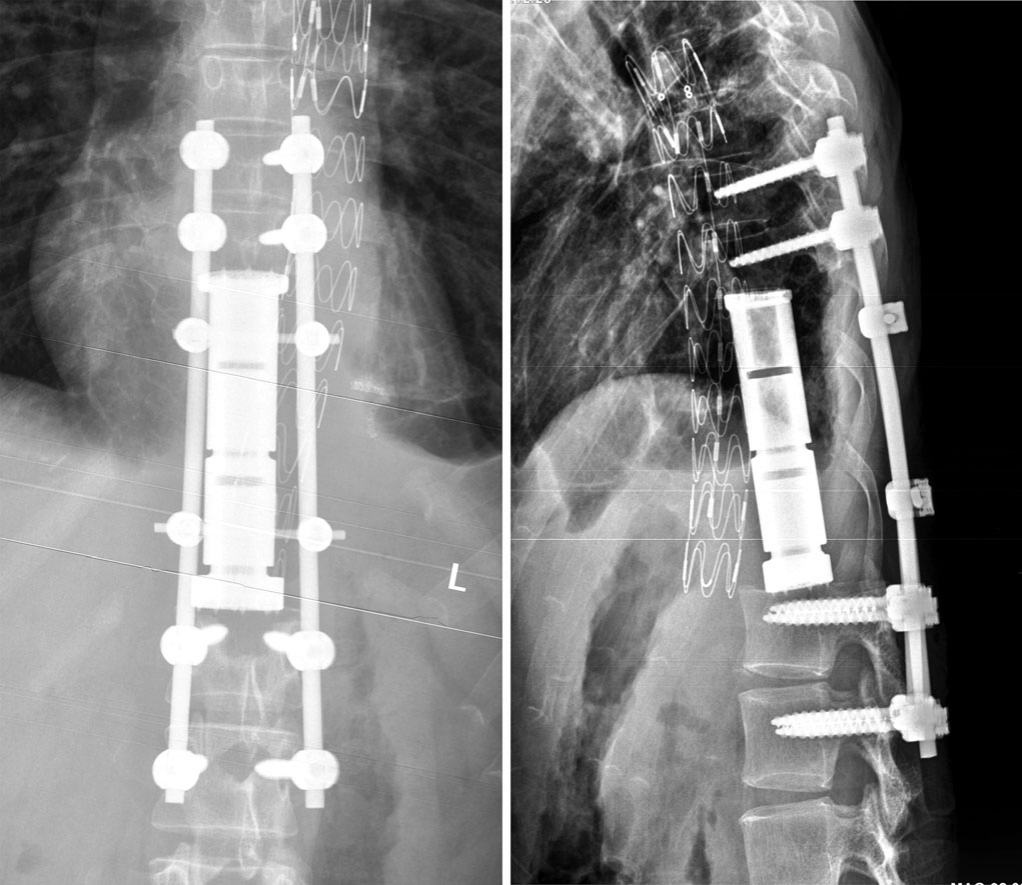
A patient (No. 16) with spinal GCT underwent TES and reconstruction with the 3D‐printed modular vertebral prosthesis. Postoperative plain radiograph.

**Fig. 5:**
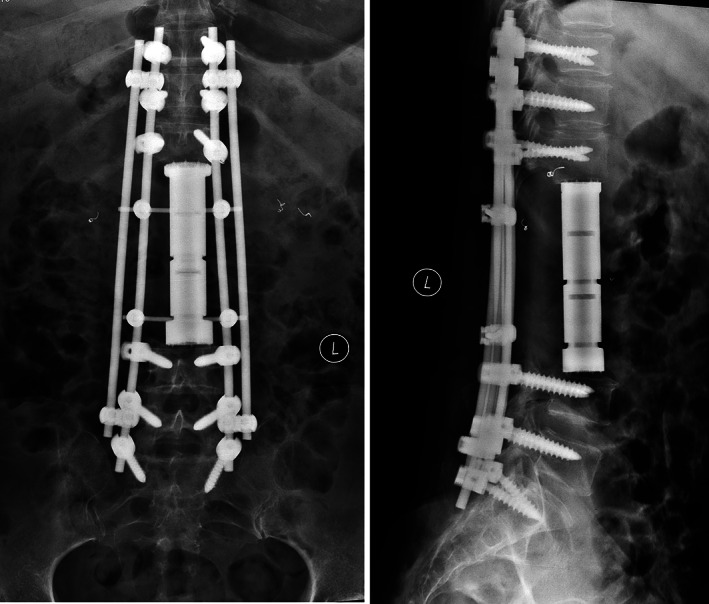
A patient (No. 21) with spinal MPNS underwent TES and reconstruction with the 3D‐printed modular vertebral prosthesis. Postoperative plain radiograph.

All operations were performed according to seven types of spinal en bloc resections described by Boriani^17^. Single posterior approach and combined approach were adopted in 11 and 16 patients, respectively. Posterior instrumentation with 2 or 3 levels above and below the resected vertebrae was carried out in all patients. After completely removed involved vertebrae (Figs 2 and 3), the length and diameter of the resected vertebral bodies were measured. A 3D‐printed modular vertebral prosthesis which had most similar size and curvature with resected specimen was assembled by simple hammering. Then the prosthesis, which was filled with granular bone allografts, was placed between the proximal and distal host vertebrae after removal of residual discs. Finally, compression was placed on the prosthesis by compressing the posterior rod‐screw instruments to achieve arthrodesis and mild spinal shortening (Figs 4 and 5).

After operation, the length of resected vertebrae was measured on the specimen again, and the surgical margins were evaluated by experienced pathologists. Wide or marginal margins were achieved in 18 patients, whereas en bloc intralesional margins were achieved in the other nine patients. Superior and inferior endplates were preserved in 16 patients, whereas nine patients had intact endplate on one side. Both sides of endplate were resected in two patients to achieve adequate surgical margins.

### Follow‐up

Postoperative follow‐up was carried out at 3‐month intervals. Local recurrence and survival situations were recorded.

#### Neurological Functions

Neurological functions were defined by motor and sensory function below the lesion level. Neurological functions before and after operation were evaluated using the Frankel Scale^18^, which included five different grades (A–E). Frankel A was complete paralysis with no presence of motor and sensory function below the lesion level. Frankel B was sensory only, which meant that there was some sensation present below the level of the lesion, but that the motor paralysis was complete below that level. Frankel C was motor useless, which implied that there was some motor power present below the lesion but it was of no practical use to the patient. Frankel D was motor useful, which meant that there was useful motor power below the level of the lesion. Frankel E was free of neurological symptoms.

#### Complications

Complications, such as dural tear, dysesthesia, and infection, were recorded and categorized into major or minor complications according to the McDonnell classification^19^. The correlation of recorded events to the presented reconstructive technique was thoroughly discussed. Prosthesis subsidence into the adjacent vertebral body was measured through follow‐up X‐ray films and computed tomography.

### Statistical Methods

All analyses were performed using SPSS version 18.0 (SPSS, Inc., Chicago, IL). Descriptive statistics were used to analyze the demographic data and clinical outcomes. Data forms included mean, standard deviation, and range.

## Results

### Demographic Data

Under general anesthesia, all patients received TES (Table 2) with an average operative time of 639 (range, 210–1650) min, and the mean blood loss during operation was 4.1 (range, 0.8–13.3) L. Twenty‐two patients have been transferred to ICU for a mean time of 3.2 (range, 0–6) d. Except for one patient who died of pulmonary infection during the perioperative period, all 26 patients had regular follow‐up with a mean time of 22 (range, 12–41) months. At the latest follow‐up, three patients died of disease, eight patients were alive with disease, and 15 patients had no evidence of disease. Local recurrence (19.2%) occurred in two patients with intralesional margin and three patients with marginal margin, respectively.

**TABLE 2:** Patient's surgical data

No.	Approach	Surgical time (min)	Blood loss (L)	ICU (d)	Surgical margins	Length of specimen (mm)	Length of prosthesis (mm)	Subsidence (mm)	Complications	
									Major	Minor
1	P	660	3.7	2	Wide	50	48	0	None	Dural tear
2	PA	480	8.4	11	Intralesional	84	84	‐	Pneumonia; TTN > 7 days	MPT
3	P	300	2.5	‐	Intralesional	48	45	1	None	None
4	P	360	4.0	‐	Intralesional	70	72	‐	None	None
5	AP	600	5.6	8	Marginal	82	80	0	Pneumonia; TTN > 7 days	None
6	AP	690	2.6	1	Marginal	68	65	1	None	Dural tear
7	P	390	0.8	1	Marginal	56	55	1	None	None
8	P	690	3.9	1	Marginal	53	50	2	None	None
9	P	600	2.5	1	Marginal	101	90	2	None	None
10	P	600	1.5	‐	Intralesional	40	40	0	None	None
11	AP	650	3.3	1	Marginal	63	60	1	Monoplegia	Dural tear
12	AP	620	7	3	Intralesional	45	45	2	TTN > 7 days	Dural tear
13	AP	600	2.2	3	Marginal	55	56	0	None	TTN 3–7 days
14	AP	390	2.2	3	Wide	72	80	0	None	Dural tear
15	P	210	3.2	3	Wide	45	44	0	None	None
16	AP	650	1.6	1	Marginal	108	90	0	Pneumonia	None
17	P	240	1.6	‐	Intralesional	42	40	0	None	None
18	AP	780	5.8	1	Intralesional	70	70	1	None	None
19	AP	1650	12.6	13	Marginal	114	105	0	PPI/TTN > 7 days/Monoplegia	Dural tear
20	AP	1330	13.3	16	Wide	142	132	3	PPI/TTN > 7 days/Wound infection/IGV/Monoplegia	MPT
21	AP	810	3.2	1	Marginal	85	85	0	None	None
22	P	300	2.2	‐	Intralesional	60	56	4	None	Dural tear
23	AP	1030	2.5	3	Marginal	110	107	0	None	TTN > 3 days/Dural tear
24	AP	1050	1.7	8	Marginal	45	45	0	PPI; TTN > 7 days; Monoplegia	Dural tear/Transient confusion
25	AP	900	5.0	6	Wide	71	68	0	IGV	None
26	AP	600	1.8	1	Marginal	100	90	0	None	None
27	P	300	5.0	1	Intralesional	50	45	0	None	None

AP, Combined anterior and posterior approach; IGV, Injury of great vessel; MPT, Multiple postoperative transfusions; P, Posterior approach; PPI, Prolonged postoperative intubation; TTN, Thoracostomy tube needed.

### Clinical Outcomes

The average length of resected thoracolumbar vertebrae was 71.4 ± 26.5 mm (range, 40–142 mm), whereas the height of reconstructive 3D‐printed modular prostheses was between 40 and 132 mm with a mean of 68.4 ± 23.9 mm. In 26 patients with minimum follow‐up of more than 1 year, no evidence of internal fixation failure or dislocation of vertebral prosthesis was found. Prosthetic subsidence into the adjacent vertebral bodies occurred in 10 patients with a mean of 1.8 ± 1.0 mm (range, 1–4 mm). The subsidence was seen at proximal end in two patients, distal end in four patients, and both ends in four patients. Subsidence of more than 2 mm occurred in two patients who had no symptoms and related internal fixation failures. The reasons were concluded as tumor local recurrence and 6 levels of spondylectomy, respectively.

### Neurological Function

Before their surgery, five patients presented with neurological deficits (two with Frankel A, two with Frankel C, and one with Frankel D). Three patients and two patients with Frankel E before surgery developed postoperative neurological symptoms likened to Frankel D and Frankel C, respectively. All patients got fully recovery at 3 or 6 months after operation. At the latest follow‐up, in 23 alive patients, 19 patients can walk independently, and two patients can achieve outdoor activities by walking aid.

### Complications

A total of 18 major complications and 14 minor complications were found in 15 patients. Pneumonia or respiratory failure necessity reintubation occurred in five patients and led to death in one patient at 11 days after surgery. Six patients had thoracostomy tube insertion for more than 7 days post‐operation. Wound infection occurred in one patient and healed after debridement. Two patients suffered from intraoperative injury of aorta which caused massive bleeding. The aorta was repaired in one patient and replaced with a prosthetic vascular graft in another one. Monoplegia of upper limb occurred in one patient whose C_8_ and T_1_ nerve roots on one side were sacrificed. One patient with 5 levels and another with 6 levels of spondylectomy went through postoperative neurological deterioration (Grade 2 power in bilateral lower limbs) which fully recovered at the final follow‐up. The most frequent minor complication among nine patients was dural tear and cerebrospinal fluid leakage.

## Discussion

For TES that completely disrupts spinal connection, effective anterior column reconstruction is needed, especially in patients with multilevel resection. An optimal reconstructive method should be suitable for different circumstances, provide instant stable fixation, and achieve final osseous fusion with low complication rate. Various techniques including autografts, allografts, mesh cages, stackable carbon cages, and 3D‐printed custom‐made prostheses have been adopted^8^, ^9^, ^13^. In recent years, we used a 3D‐printed modular vertebral prosthesis for reconstruction after TES. The purpose of this study is to evaluate the preliminary results of this prosthetic reconstruction after multilevel thoracolumbar TES.

### Is this Prosthesis Suitable for Different Lengths of Reconstruction?

The 3D‐printed modular vertebral prosthesis is suitable for different length of thoracolumbar spine reconstructions, especially in multilevel TES which creates severe spinal instability. For single‐level vertebral defects, titanium mesh cages filled with auto/ allograft granular bone or expandable cages are sufficient for anterior column reconstruction^9^. With aggressive multilevel TES, a large spinal column defect is created and needs more effective anterior column reconstructions. In a series^6^ of nine patients who received a minimum 4‐level en bloc spondylectomy, the mean size of resected specimen was 8.7 × 6.4 × 4.6 cm. In our study, the length of resected specimen was between 40 and 142 mm. For such extensive spine column reconstruction, only few optimal methods could be expected. It has been suggested that structural bone graft should be used when spanning a defect greater than two vertebral bodies^9^. However, the donor site morbidity and time consumption of vascular anastomosis still need to be considered. The expandable cage has the disadvantages of insufficient amount of bone graft and limitation of use in extensive spinal column reconstruction. The use of modular carbon stackable cages seems to be an excellent option for patients with multilevel TES^7^, ^8^. The advantages include fusion between carbon fiber and bone interface, low scattering in imaging, and convenience for postoperative radiation. The only concern is about its expensive price, especially in large‐scale reconstructions. Custom‐made 3D‐printed vertebral prosthetic replacement^13^, ^14^, ^15^ is another reconstructive option. However, these custom‐made prostheses have drawbacks including a period of waiting time for production and difficulty of reconstruction in cases of a mismatch between the resected specimen and the prosthesis. The authors suggest an alternative reconstruction option to be always available, in case of intraoperative change in surgical plan. In our study, variable combinations of length, diameter, and degree of curvature to fit different needs of reconstruction can be achieved by using the 3D‐printed modular vertebral prosthesis assembled during operation. As the maximum length of this modular prosthesis is 200 mm, the spinal reconstruction had been successfully carried out even in a patient with 6‐level TES.

### Does this Prosthesis Have Fewer Mechanical Complications?

The 3D‐printed modular vertebral prosthesis has fewer mechanical complications. Instrument failure after TES, which is attributed to both unstable fixation of anterior column reconstruction in the early stage and subsidence to adjacent vertebrae in the later period, is not uncommon. Its rate can be as high as 40%^4^, and 7.7% of patients received revision surgery^9^. Studies^20^, ^21^ have revealed that cage subsidence is more common after multilevel corpectomy. Mesh cages filled with granular bone grafts have a high incidence of subsidence, which was observed in 50% of patients who receive TES at 3 or more levels^21^. Expendable titanium cage is deemed to have a low hardware failure rate for reconstruction in patients with vertebral body resection. In a study by Viswanathan *et al*.^10^ that including 95 spine tumor patients, only three patients experienced internal fixation failure; the other 12 patients demonstrated subsidence of greater than 1 mm, but none required operative revision. However, the majority of patients in their study had single‐level vertebrectomy, while only 20 patients received multilevel resection (19 two‐level and one three‐level). 3D‐printed custom‐made prosthesis also has a high incidence of asymptomatic subsidence in reconstruction after TES. In a study^13^ of 13 patients (10 single‐level and three double‐level), subsidence occurred in all cases with a mean degree of nearly 2.8 mm at both proximal and distal sides. Fortunately, revision of the construct was needed in only one patient. In our series, although most patients experienced major complications related to combined approach and multilevel surgical procedures^5^, no mechanical failure occurred with this 3D‐printed modular vertebral prosthesis. Only two patients had more than 2 mm subsidence and all internal fixations were intact. The prosthetic end plates with porous surface and tiny sharp conical projections, which provide early fixation and eventually osseous union, may reduce the rate of subsidence and internal fixation failure.

### Can this Prosthesis Provide a Stable Environment to Maintain or Rehabilitate Patients' Neurological Function?

The 3D‐printed modular vertebral prosthesis can provide a stable environment to maintain or recover patients' neurological functions. One of the disaster complications with multilevel TES is spinal cord ischemia attributable to the ligation of segmental vessels including the Adamkiewicz artery. Studies^22^, ^23^ in animal models have shown that bilateral ligation of segmental arteries at 3 levels did not damage spinal cord function, whereas those greater than 4 levels increased the risk for spinal cord ischemia. Therefore, reduced blood supply for spinal cord is often encountered in multilevel TES. In our series, five of 26 patients suffered from postoperative neurofunctional deterioration and eventually recovered. For the increase of blood flow in the spinal cord, an average rate of 12.3% of spinal shortening is recommended in anterior column reconstruction after multilevel TES^21^. With the help of this 3D‐printed modular vertebral prosthesis, which provided accurate length changes with a minimum interval of 2.5 mm, proper spinal shortening was achieved in our patients, and the neurological functions of the patients were recovered or preserved.

### Limitations

This retrospective study has limitations. First, the number of patients is relatively small and the follow‐up period is short. Second, osseous fusion between the 3D‐printed prosthesis and the host bone, which is hardly revealed even on postoperative computed tomography, has not been evaluated. Third, there is lack of a control group to verify whether 3D‐printed modular prosthesis is superior or inferior to alternatives. However, owing to the rarity of primary malignant spine tumors and limited indications for TES, having an initial impression followed by long‐term follow‐up is worthwhile.

### Conclusion

The preliminary results of this study on 3D‐printed modular vertebral prosthesis for spinal reconstruction after multilevel thoracolumbar TES showed that the prosthesis is suitable for different lengths of anterior column reconstruction with less mechanical complications, and can provide a stable environment to maintain or rehabilitate patients' neurological function in short‐term follow‐up. More patients with long follow‐up time will be needed to prove its efficiency.
